# How much change is enough? Evidence from a longitudinal study on depression in UK primary care

**DOI:** 10.1017/S0033291720003700

**Published:** 2022-07

**Authors:** Daphne Kounali, Katherine S. Button, Gemma Lewis, Simon Gilbody, David Kessler, Ricardo Araya, Larisa Duffy, Paul Lanham, Tim J. Peters, Nicola Wiles, Glyn Lewis

**Affiliations:** 1 Bristol Medical School, University of Bristol, Bristol, UK; 2 Department of Psychology, University of Bath, UK; 3 Division of Psychiatry, University College London, London, UK; 4 Department of Health Sciences, University of York, York, UK; 5 Institute of Psychiatry, Psychology and Neuroscience, King's College London, London, UK

**Keywords:** Baseline severity depression, BDI-II, beta-regression, depression, GAD-7, minimal clinically important difference, PHQ-9, primary care

## Abstract

**Background:**

The Patient Health Questionnaire (PHQ-9), the Beck Depression Inventory (BDI-II) and the Generalised Anxiety Disorder Assessment (GAD-7) are widely used in the evaluation of interventions for depression and anxiety. The smallest reduction in depressive symptoms that matter to patients is known as the Minimum Clinically Important Difference (MCID). Little empirical study of the MCID for these scales exists.

**Methods:**

A prospective cohort of 400 patients in UK primary care were interviewed on four occasions, 2 weeks apart. At each time point, participants completed all three questionnaires and a ‘global rating of change’ scale (GRS). MCID estimation relied on estimated changes in symptoms according to reported improvement on the GRS scale, stratified by baseline severity on the Clinical Interview Schedule (CIS-R).

**Results:**

For moderate baseline severity, those who reported improvement on the GRS had a reduction of 21% (95% confidence interval (CI) −26.7 to −14.9) on the PHQ-9; 23% (95% CI −27.8 to −18.0) on the BDI-II and 26.8% (95% CI −33.5 to −20.1) on the GAD-7. The corresponding threshold scores below which participants were more likely to report improvement were −1.7, −3.5 and −1.5 points on the PHQ-9, BDI-II and GAD-7, respectively. Patients with milder symptoms require much larger reductions as percentage of their baseline to endorse improvement.

**Conclusions:**

An MCID representing 20% reduction of scores in these scales, is a useful guide for patients with moderately severe symptoms. If treatment had the same effect on patients irrespective of baseline severity, those with low symptoms are unlikely to notice a benefit.

**Funding:**

Funding. National Institute for Health Research.

## Introduction

Depression is a common reason for consultation in primary care (McManus et al., 2014) and a major public health problem. Clinicians are faced with the difficulty of making treatment recommendations for patients they see in primary care based upon evidence from studies that used assessments for depressive symptoms that were developed primarily for research purposes. Deciding what constitutes a clinically important treatment effect for those research assessments is therefore essential for interpreting the results of clinical research and designing randomised trials.

The minimum clinically important difference (MCID) provides a measure of the smallest change in an outcome that is perceived as important to patients. This must be determined, to understand the clinical utility of therapies intended to improve subjective outcomes.

The UK National Institute for Health and Care Excellence (NICE) proposed a reduction of three points on the Hamilton Depression Rating Scale as clinically important, but this was based solely on the opinion of an expert group (Kendrick and Pilling, 2012). Others have used approaches that rely upon the error of measurement of the scales. Clinician ratings of improvement have also been frequently used in depression research but none of these approaches incorporates the patient's perspective (Leucht et al., 2013).

Clinicians and policy makers give more emphasis on patients’ perspectives in the evaluation of interventions and public health policies. It is therefore important to establish an MCID anchored in the experiences and perceptions of patients (Tubach et al., 2012; Hinman et al., 2014). In previous work, we have investigated the MCID for the Beck Depression Inventory (BDI-II) from the perspective of the patient (Button et al., 2015). Using a Global Rating of Change Scale (GRS), patients were asked whether they felt better, the same or worse since they were last seen and the MCID was calculated as the minimum change in depression scores associated with reporting feeling ‘better’.

The current study further develops the previous approach. The aim was to estimate the MCID for the BDI-II, PHQ-9 and GAD-7 scales. It studies a sample of primary care patients who have been consulting about symptoms of depression and anxiety with broad inclusion criteria to better reflect the population seeking help and including patients with low levels of symptoms. We planned to simply observe spontaneous changes in patients’ scores as patients will be at all stages of their treatment and we did not provide any intervention ourselves.

We have extended our previous work in several other ways. Firstly, we include the PHQ-9 and GAD-7 that are frequently used in research and are the standard outcome measures in Improving Access to Psychological Therapies (IAPT) services. Secondly, we have used a more flexible method of analysis to allow comparisons between those reporting improvements against those reporting ‘feeling the same’, over multiple waves. Our previous research merged the latter group with those ‘feeling worse’. Finally, we have taken three different approaches to estimate the MCID: the mean change for those ‘feeling better’, the mean difference in change between ‘feeling better’ and ‘feeling the same’, and the threshold value below which participants are more likely to report ‘feeling better’ rather than report ‘feeling the same’.

## Method

### Participants

The sample was recruited from primary care surgeries in three UK sites (Bristol, Liverpool, and York) between February 2013 and April 2014. This study was part of the PANDA programme (NIHR programme ‘What are the indications for Prescribing ANtiDepressAnts that will lead to clinical benefit?’; NIHR Programme Grant: RP PG 0610 10048). One of the primary objectives of this element of the programme was to estimate the MCID for measures of depression by assembling a pragmatic and contemporary cohort of patients seeking help in primary care with a broad range of depression symptom severity. As anxiety symptoms are often co-morbid with depression and no NICE guideline addresses such presentations, the study also collected data on a measure of generalised anxiety, the GAD-7, enabling us to explore the MCID for this measure (Kendrick and Pilling, 2012).

Computerised records at collaborating general practices at each site were searched to identify people who had reported depressive episodes, depressed mood, depressive symptoms, or a major depressive episode in the past year. Individuals were included if they were aged between 18 and 74 years, treated or not treated with antidepressants, and referred or not referred to IAPT services. We excluded people who: were diagnosed with bipolar disorder, psychosis, or an eating disorder; had alcohol or substance use problems; were unable to complete study questionnaires; or were 30 weeks or more pregnant. Overall, 7721 patients were sent an information letter in the post and 1470 (19%) replied. Of these, 821 were willing to be contacted, 23 (3%) of whom were ineligible. The remaining 798 were contacted to arrange an interview, and 563 consented to take part in the cohort study.

Data on our measures were collected at four time points, each approximately 2 weeks apart so that participants were in the study for 6 weeks between baseline or time 1 and time 4. At time one, 559 people provided data (four could not be contacted), with corresponding figures at follow-ups two, three and four of 476 (85%), 443 (79%) and 430 (77%) respectively. In total, 400 (72%) participants provided data at each of the four follow-ups and were included in our analyses. Participants with missing data at one or more follow-ups were excluded. Participation and attrition rates are similar to those encountered in pragmatic clinical trials in primary care (Richards et al., 2016; Lewis et al., 2019). We found no evidence of a relationship between follow-up rates and observed CIS-R scores or depression and anxiety symptoms at baseline.

Interviews were conducted at the participant's home or GP surgery. All participants provided written informed consent and ethical approval was obtained from National Research Ethics Service Committee South West-Central Bristol (REC No:12/SW/0267). The authors assert that all procedures contributing to this work comply with the ethical standards of the relevant national and institutional committees on human experimentation and with the Helsinki Declaration of 1975, as revised in 2008.

The recruitment of participants is irrespective of the treatment they are receiving and the duration of their treatment. We relied therefore upon spontaneous changes in symptoms for our analyses.

## Measures

### Beck Depression Inventory–II (BDI-II)

The BDI-II (Beck et al., 1996) is a self-report measure of the severity of depressive symptoms, consisting of 21 items, each assessed using a 4-point scale ranging from 0 to 3. Possible scores range from 0 to 63. Higher scores indicate greater severity of depressive symptoms. Participants were asked about the previous 2 weeks.

### Patient Health Questionnaire (PHQ-9)

The PHQ-9 (Kroenke and Spitzer, 2002) is a self-report measure of the severity of depressive symptoms, consisting of nine items each with a 4-point scale ranging from ‘Not at all’ (0) to ‘Nearly every day’ (3). Possible scores range from 0 to 27 and higher scores indicate greater severity of depressive symptoms. The PHQ-9 asked about the previous 2 weeks.

### Anxiety

The Generalised Anxiety Disorder Assessment (GAD-7) (Spitzer et al., 2006) was used to measure anxiety at each time point. The GAD-7 is a self-report measure of generalised anxiety symptoms consisting of seven items, each assessed using a 4-point scale ranging from ‘Not at all’ (0) to ‘Nearly every day’ (3). Possible scores range from 0 to 21. Higher scores indicate greater severity of anxiety and questions were asked about the previous 2 weeks.

### Global rating of change scale

The global rating of change scale is a self-report measure of subjective well-being over time, asking participants: ‘Compared to when we last saw you 2 weeks ago, how your moods and feelings have changed?’. The five possible responses were: ‘I feel a lot better’ (1), ‘I feel slightly better’ (2), ‘I feel about the same’ (3), ‘I feel slightly worse’ (4), ‘I feel a lot worse’ (5). Participants completed two global rating of change scales (separated by other questionnaires) at each time point, to assess reliability (Kamper et al., 2009; Robinson et al., 2017).

### Clinical Interview Schedule-Revised (CIS-R)

The CIS-R (Lewis et al., 1992) is a fully structured self-administered computerised assessment of common mental disorders that have been extensively used in community samples. Participants were assessed using the CIS-R at baseline or time 1 only. The thresholds used (0-11/12-19/20+) were those pre-specified in the protocol for the subsequent PANDA trial (Salaminios et al., 2017).

### Demographics

Demographic variables were measured at baseline using a self-administered computerised assessment. These were age, gender, ethnicity, employment status, financial status and education level.

### Current antidepressant use

A short self-report measure was used to assess current medication use at each time point. Participants were asked whether they were currently taking antidepressants.

## Statistical analyses

### Accounting for baseline severity

We previously found that MCID on the BDI-II in absolute terms varied according to baseline severity, with larger MCID estimates at higher levels of severity (Button et al., 2015). Stratification by baseline CIS-R groupings is motivated by the desire to examine the potential dependence of the MCID on baseline severity. CIS-R stratification is attractive statistically as it is highly correlated with all measures and for this reason, increase precision in estimates of change. In the current study, we also noted that the relationship between the GRS and severity on the three measures was different for participants with low (⩽11), medium (12–19) and high (⩾20) total scores on CIS-R completed at time 1. We also found that the mean initial PHQ-9 score in the group reporting ‘feeling the same’ is lower than in those reporting ‘feeling better’ when baseline severity is low (CIS-R⩽11) (Table 1). In contrast, in the high (CIS-R > 20) the mean initial PHQ-9 score was lower in those reporting ‘feeling better’ compared to those reporting ‘feeling the same’. These patterns were similar for all outcomes (Tables 2 and 3).

**Table 1.** Estimate initial and change in PHQ9 score (previous 2 weeks) according to patient reported Global ratings and time 1 CIS-R

Global Rating Scale	*N*^(*)^	Feeling better			*N*^(*)^	Feeling same			*N*^(*)^	Feeling worse		
		Mean	95% CI			Mean	95% CI			Mean	95% CI	
Baseline CIS-R		Initial PHQ9										
≤11	36	4.15	(3.07	5.39)	78	2.66	(2.15	3.26)	9	6.08	(3.38	9.59)
12–19	25	7.97	(6.08	10.17)	58	8.75	(7.51	10.11)	9	11.06	(7.49	15.25)
20+	32	12.20	(9.91	14.60)	117	15.01	(13.98	16.07)	36	17.23	(15.11	19.09)
		Change in previous 2 weeks										
≤11		−1.00	(−1.45	−0.63)		−0.28	(−0.48	−0.09)		0.75	(0.15	1.41)
12–19		−1.66	(−2.28	−1.11)		−0.83	(−1.33	−0.32)		0.45	(−0.20	1.12)
20+		−2.38	(−2.85	−1.88)		−0.66	(−1.05	−0.26)		0.27	(−0.15	0.72)
		Change in previous weeks as a proportion of initial PHQ9 score										
≤11		−0.24	(−0.31	−0.17)		−0.10	(−0.17	−0.03)		0.13	(0.03	0.24)
12–19		−0.21	(−0.27	−0.15)		−0.09	(−0.15	−0.04)		0.04	(−0.02	0.10)
20+		−0.20	(−0.24	−0.15)		−0.04	(−0.07	−0.02)		0.02	(−0.01	0.04)

(*) *N* denotes sample size at baseline for each group. Detailed account of the number of participants contributing to the estimates in each visit is given in Appendix 2 Figure S2.1.

**Table 2.** Estimate initial and change in BDI-II score (previous 2 weeks) according to patient reported Global ratings and time 1 CIS-R

Global Rating Scale	Feeling better				Feeling same				Feeling worse			
	*N*^(*)^	Mean	95% CI		*N*^(*)^	Mean	95% CI		*N*^(*)^	Mean	95% CI	
Baseline CIS-R		Initial BDI										
≤11	36	9.67	(7.52	12.02)	78	6.24	(5.29	7.27)	9	11.74	(7.07	17.37)
12–19	25	14.68	(11.32	18.78)	58	15.94	(13.69	18.26)	9	16.54	(10.99	22.76)
20+	32	22.25	(18.80	26.07)	117	26.99	(24.77	29.13)	36	31.68	(28.05	35.50)
		Change per 2 weeks										
≤11		−2.97	(−3.89	−2.19)		−1.28	(−1.67	−0.93)		0.11	(−0.79	1.02)
12–19		−3.36	(−4.46	−2.49)		−1.61	(−2.31	−0.93)		−0.12	(−1.00	0.78)
20+		−4.32	(−5.16	−3.51)		−1.57	(−2.20	−0.94)		0.14	(−0.61	0.93)
		Change per 2 weeks as a percentage of initial BDI score										
≤11		−0.31	(−0.36	−0.25)		−0.21	(−0.25	−0.16)		0.01	(−0.07	0.09)
12–19		−0.23	(−0.28	−0.18)		−0.10	(−0.15	−0.06)		−0.01	(−0.06	0.05)
20+		−0.20	(−0.23	−0.16)		−0.06	(−0.08	−0.03)		0.01	(−0.02	0.03)

(*) *N* denotes sample size at baseline for each group. Detailed account of the number of participants contributing to the estimates in each visit is given in Appendix 2 Figure S2.1.

**Table 3.** Estimate initial and change in GAD-7 score (previous 2 weeks) according to patient reported Global ratings and time 1 CISR

Global Rating Scale	*N*^(*)^	Feeling better			*N*^(*)^	Feeling same			*N*^(*)^	Feeling worse		
		Mean	95% CI			Mean	95% CI			Mean	95% CI	
Baseline CIS-R		Initial GAD-7										
≤11	36	3.05	(2.26	4.01)	78	1.92	(1.51	2.37)	9	5.24	(3.07	7.88)
12–19	25	5.62	(4.14	7.26)	58	6.23	(5.25	7.33)	9	5.24	(3.02	7.82)
20+	32	9.02	(7.37	10.93)	117	11.12	(10.18	11.98)	36	13.97	(12.38	15.39)
		Change per 2 weeks										
≤11		−0.81	(−1.18	−0.50)		−0.27	(−0.44	−0.11)		0.86	(0.27	1.54)
12–19		−1.50	(−2.04	−1.03)		−0.53	(−0.92	−0.11)		0.00	(−0.47	0.50)
20+		−1.56	(−1.96	−1.16)		−0.42	(−0.76	−0.08)		0.67	(0.28	1.06)
		Change per 2 weeks as a percentage of baseline GAD7 score										
≤11		−0.26	(−0.34	−0.19)		−0.14	(−0.21	−0.06)		0.17	(0.05	0.30)
12–19		−0.27	(−0.34	−0.20)		−0.09	(−0.15	−0.02)		0.00	(−0.09	0.10)
20+		−0.17	(−0.22	−0.13)		−0.04	(−0.07	−0.01)		0.05	(0.02	0.08)

(*) *N* denotes sample size at baseline for each group. Detailed account of the number of participants contributing to the estimates in each visit is given in Appendix 2 Figure S2.1.

The group of patients who report feeling the same is a mixture of people who have high and low baseline scores. The reason why those reporting ‘feeling the same’ at low baseline severity did not experience any symptom change could be that they are not currently depressed. This is supported by our observations. Among those reporting ‘feeling the same’ at baseline only 54% were on current medication in the mild severity group whilst among those reporting ‘feeling better’ 72% were on current medication. As baseline severity increases, it becomes more likely that this group report no change because of lack of improvement of their symptoms. Thus, baseline CIS-R stratification provides a clinical context for understanding baseline variability in scores. Using the CIS-R also conferred the advantage of providing a measure of baseline severity independent of the scales of interest.

### Reliability of the global rating of change scale (GRS)

Reliability of the Global Rating of change scale was quantified using the two repeated assessments completed by the patient within each period. Levels of agreement for the GRS were estimated via the (unweighted) Kappa coefficient (Landis and Koch., 1977). We also assessed GRS reliability using the intra-class correlation coefficient (Skrondal and Rabe-Hesketh. 2004). These calculations were conducted using Stata version 15 (StataCorp, 2015).

### Change in BDI-II, PHQ-9 and GAD-7 scores – modelling

We used Bayesian hierarchical regression models to estimate the changes in symptom scores measured by the three scales (BDI-II and PHQ-9 and GAD-7) and over three waves (times 1 and 2, 2 and 3 and 3 and 4). These estimates were allowed to vary according to GRS groupings and baseline CIS-R score (Zimprich, 2010; Verkuilen and M, 2012). Estimates of changes were based on their respective contemporaneous GRS ratings at each wave. The baseline GRS informed estimates of initial scores specific to each CIS-R group. Our estimates of MCID were based upon the GRS at time 2, 3 and 4 that were combined with changes in scores between time 1 and 2, 2 and 3 and 3 and 4, respectively. We can then arrive at estimates across those time periods of the initial score and changes in score according to GRS category.

We carried out comparisons of different models using various distributional assumptions and link functions and found the beta-regression to perform best in terms of overall model fit criteria (DIC) (Spiegelhalter et al., 2002).

A detailed description of the model specifics is provided in online Appendix 1. We carried out model fitting, model comparisons and post-estimation calculations using the WinBUGS statistical software (Spiegelhalter et al., 2007).

Given the small sample sizes in some GRS response options, these were amalgamated as follows: ‘I feel a lot better’ (1) and ‘I feel slightly better’ (2) under the revised category ‘Feeling better’; ‘I feel slightly worse’ (4) and ‘I feel a lot worse’ (5) under the revised category: ‘Feeling worse’. This amalgamation did not confer noticeable loss of information for the comparisons of interest, since 72% of responses below GRS level 2 were endorsing level 2 and 77% of response above level 4 were endorsing level 4 without considering any other breakdown in baseline severity (Appendix 1). We have included the data on all three GRS categories for the sake of completeness.

We express differences in terms of proportional as well as absolute scores using standard post-estimation calculations. The variability in the distribution of change in the different groups was also estimated.

### Receiver operating characteristic (ROC) analysis

We estimated the threshold value of change that corresponds to the maximum improvement in sensitivity over chance.

The ROC parameters required for the derivation of the MCID and sensitivity and specificity determination were based on calculations for functions of the parameters estimated from the above regression models, assuming approximate normality (Appendix 1).

In Table S1.1 (Appendix 1), we present uncertainty estimates of the sensitivity and specificity at the optimal threshold. Statistics relevant to the determination of the optimal threshold are presented in Table S1.2, along with standardised estimates of change in Table S1.3.

## Results

### Sample characteristics

We restricted the analysis to the 400 patients who had complete data for all four time points. No baseline differences between excluded and included patients were apparent in the outcomes under study or their demographics. Demographic and clinical characteristics are shown in Table S2.1 (Appendix 2). Participants were aged 17–71 years (mean = 48.7), and the majority were female, white, married, and employed. Roughly a third of participants had completed higher education. Just under half of participants met ICD-10 criteria for major depressive disorder at baseline. The vast majority reported using antidepressants at each time point.

Descriptive statistics of the distribution of GRS scale over time overall as well as stratified by CIS-R are presented in Appendix 2 (Table S2.2, Figure S2.1). The CIS-R score was strongly associated with all self-rated scales *BDI-II, PHQ-9 and GAD-7*, with correlations at baseline of 0.82, 0.78 and 0.77, respectively. There were no changes in GRS scores over time beyond that expected by chance.

### Test-retest reliability of the GRS

Levels of agreement for the GRS were found to be substantial or excellent, with kappa values of 0.73, 0.84, 0.86 and 0.81 for baseline, first, second and third visits, respectively. The intraclass correlation coefficients were: 0.95 (95% CI 0.94–0.96) at baseline; 0.98 (0.97–0.99) at the first visit; 0.92 (0.90–0.94) at the second; and 0.99 (0.98–0.99) at the third.

### Change in BDI-II, PHQ-9 and GAD-7 over time for each grouping of the global rating of change (GRS) scale

In Table 1, we present estimated mean initial levels and changes in mean scores in both absolute and proportional terms for each CIS-R severity group and GRS group on the PHQ-9. Tables 2 and 3 provide the same estimates for the BDI-II and GAD-7. The initial scores vary depending upon the CIS-R groups. The changes required for people to report ‘feeling better’ increase with baseline severity (Figures S3.1-S3.3, Appendix 3). It is also noteworthy that the increases seen for those ‘feeling worse’ were not as large as the reductions in those reporting ‘feeling better’.

No differences in the estimated percentage changes for those reporting ‘feeling better’ was found across CIS-R severity groups, for all outcomes (Tables 1–3).

Participants who reported ‘feeling the same’, also experienced reductions in score on all outcomes, though smaller than those who reported ‘feeling better’. In Table 4 we have estimated the difference in the changes reported by those who report ‘feeling better’ and those who report ‘feeling the same’, in absolute scores as well as a percentage of their respective baseline scores. In general, the differences between ‘feeling better’ and the same became larger as the CIS-R severity increased.

**Table 4.** Estimated difference in change between the group reporting feeling better and the group reporting feeling the same in absolute scores and % from their respective initial scores for PHQ9, BDI-II and GAD-7 scales

Baseline Severity	CIS-R 0–11			CIS-R 12–19			CIS-R 20+		
	Mean	95% CI		Mean	95% CI		Mean	95% CI	
Outcome	Difference in change								
PHQ9	−0.73	(−1.13	−0.40)	−0.85	(−1.45	−0.31)	−1.70	(−2.18	−1.24)
BDI	−1.66	(−2.54	−0.89)	−1.76	(−2.74	−0.79)	−2.77	(−3.61	−1.94)
GAD-7	−0.54	(−0.88	−0.24)	−0.99	(−1.53	−0.49)	−1.15	(−1.57	−0.72)
	Difference in % change								
PHQ9	−0.14	(−0.22	−0.07)	−0.12	(−0.18	−0.06)	−0.15	(−0.19	−0.11)
BDI	−0.10	(−0.16	−0.04)	−0.13	(−0.18	−0.08)	−0.14	(−0.17	−0.10)
GAD-7	−0.12	(−0.21	−0.04)	−0.18	(−0.26	−0.11)	−0.14	(−0.18	−0.09)

### ROC analysis

In Table 5, we present our estimates from the ROC analysis. The ROC analysis selects the optimal threshold below which participants are more likely to report ‘feeling better’ rather than ‘feeling the same’. The mean change in the group reporting ‘feeling better’ (see Tables 1–3) is a good approximation for the optimal threshold when the baseline symptom severity is moderate and high for all three instruments. However, when the depression severity is low, the optimal threshold needs to be considerably lower than the mean change to optimise the discrimination between the two groups (Figure Appendix 2: S2.2a-S2.2c).

**Table 5.** Estimated threshold score for discriminating between feeling better and feeling the same for the PHQ9 BDI-II and GAD-7 scales according to baseline severity and related ROC parameters

Instrument Scale	Severity	Threshold score	Threshold as % of baseline	95% change as % of baseline	Spec^(1)^	Sens^(2)^	AUC^(3)^ Mean	(2.5%	97.5%)
PHQ9	≤11	−2.0	48.2	(65.1 37.1)	0.78	0.35	0.57	(0.54	0.60)
	12–19	−1.7	21.3	(27.9 16.7)	0.59	0.51	0.57	(0.53	0.62)
	20+	−2.4	19.7	(24.2 16.4)	0.63	0.52	0.61	(0.58	0.64)
BDI	≤11	−5.0	51.7	(66.6 41.6)	0.80	0.36	0.59	(0.55	0.63)
	12–19	−3.5	23.8	(30.9 18.6)	0.65	0.50	0.60	(0.55	0.65)
	20+	−4.4	19.7	(23.4 16.9)	0.65	0.51	0.61	(0.58	0.65)
GAD-7	≤11	−2.2	72.1	(97.3 54.8)	0.78	0.32	0.55	(0.53	0.59)
	12–19	−1.5	26.7	(36.2 20.7)	0.51	0.62	0.59	(0.55	0.64)
	20+	−0.8	8.9	(10.9 7.3)	0.55	0.54	0.57	(0.54	0.59)

(1) Probability (improvements/reductions smaller than MCID when feeling the same).

(2) Probability (improvements/reductions larger than MCID when feeling better).

(3) Probability the improvement (reduction) in scores for a randomly chosen patient drawn from those reporting feeling the same is smaller than for a randomly chosen person drawn from those reporting feeling better.

These results illustrate that at lower levels of depression severity participants are less good at discriminating between ‘feeling better’ and ‘feeling the same’ for all three scales. The optimal threshold was estimated at 2 points and was not greatly affected by baseline severity for the PHQ-9. The threshold score for the BDI-II was 5 points at low baseline severity – higher than the 4 points for moderate and high CIS-R. Finally, the threshold score for GAD-7 was 2 points for low and moderate CIS-R and 1 point for high CIS-R at time 1 (Table 5). The absolute change was similar across the three severity bands, but the percentage change increased in the least severe band. This is true for all measures.

At low baseline CIS-R, the sensitivity (Table 5) was 35%, 36% and 32% for PHQ-9, BDI-II and GAD-7 respectively, representing the proportion who reported they felt better and had experienced reductions larger than the threshold score. At higher baseline CIS-R, the patients who reported improvement had much higher chances (60% or more) to show reductions larger than the threshold score in all scales. The data on standard deviations and standardised change, further illustrates that the reduction in MCID at lower severity is due to reduced sensitivity to change and increased variance (Appendix 1: Table S1.2 and S1.3).

## Discussion

We have estimated the minimally clinically important difference using a patient-centred approach for three commonly used scales employed to assess depression and anxiety. We have estimated the reduction in scores during the previous 2 weeks in those who reported ‘feeling better’. We then estimated the difference between ‘feeling better’ and ‘feeling the same’ in terms of the reduction of scores.

We also formulated the problem as trying to distinguish between ‘feeling better’ and ‘feeling the same’ using ROC analysis to arrive at the threshold value that provides maximal separation and accounts for the increased variability of scores at both ends of these scales.

In the lowest severity group, average reductions experienced by those reporting ‘feeling better’ were much smaller (around 20–30%) than the optimal thresholds required to discriminate between ‘feeling better’ and ‘feeling the same’ (between 48% and 71%). This was true for all three scales. The marked increase of threshold in percentage terms is due to the increased variability of change, particularly in those feeling better at lower baseline severity and this makes discrimination more difficult. At low scores, patients will only be able to detect a relatively large proportionate change.

In our previous work, we found evidence that viewing the MCID as a proportion led to a more constant value over the severity range (Button et al., 2015). However, this was based on analyses informed by randomised controlled trials (RCTs) which excluded patients below a certain threshold score and similar distributions of baseline scores on the BDI scale. In this study with a sample with lower severity scores, it is apparent that there is still an increase in MCID in proportional terms at lower levels of severity, even if the absolute levels are relatively constant. It is perhaps unsurprising that those with low scores will find it more difficult to distinguish between ‘feeling the same’ and ‘feeling better’. The likely explanation is that the reliability of change for these scales is also dependent on the baseline.

It is striking that there are many similarities in how the different scales behave in relation to self-reported improvement. Previous meta-analytic work evaluating the relative responsiveness of eight scales (6 depression and 2 quality of life) also found little difference between scales capturing change caused by treatment (Kounali et al., 2016). The study included a broad range of different treatments and even though the absolute values of the scales differed, the pattern of results was similar and the proportionate changes seemed comparable.

### Strength and limitations

This is the first study on establishing multiple MCIDs from a large contemporary cohort drawn from a population seeking help for their symptoms in primary care in the UK. In contrast to our previous study that used data from RCTs, this sample was not selected according to severity criteria. Our approach allowed for a realistic assessment of the distribution of change, which is implicated in the determination of the optimal threshold. The results enhance our earlier work by extending it to lower severities of symptoms and to include other commonly used outcome measures, the PHQ-9 and GAD-7. In this study, as in our previous work, we are assuming that the MCID is the same irrespective of whether the change results from treatment or is spontaneous. Nevertheless, in this study, we acknowledge that we had little information on treatments and how this might have affected MCID estimates.

Despite the size of this cohort, the number with low CIS-R baseline severity who report ‘feeling better’ at baseline is still rather small (*n* = 36), so some of our estimates lacked precision. Our method also relied on the use of self-reported improvement. It remains unclear how patients’ perceptions of change can inform therapeutic significance, but it is certainly an aspect of this. Using self-reported change as a ‘gold standard’ has good face validity (Malpass et al., 2016) and qualitative findings support its use. Yet our results indicate areas where our understanding of how patients perceive and retrospectively recall change requires further research. For example, the finding that people who reported ‘feeling the same’ also had reductions in symptoms is not well understood (Robinson et al., 2017). One possible explanation is that the patient's GRS is likely to include constructs additional to those measured by the disease-specific scales, so a perfect correlation is not expected. Research in health-related quality of life has also found that retrospective measures of the patient's view of change is sensitive to change in disease-specific scales and correlates strongly with patient's satisfaction with change but is not concordant with repeated current assessments of patients’ experience of change (Fischer et al., 1999). This literature also presents evidence that those with less severe symptoms at baseline have smaller change scores over time; thus, variability in baseline severity may reduce the strength of association between change scores and the GRS (Stucki et al., 1996). It is also noteworthy that there was a marked asymmetry in this sample such that feeling worse was not associated with such large changes as ‘feeling better’. The reasons for this are unknown.

Comparative elicitation of improvement using clinician-rated scales could have been interesting. Self-administered scales are used clinically in primary care and psychological services (IAPT). Even though there is much pharmacological data using the HAMD, it is not used in routine clinical practice. Our choice of using the patient's GRS was influenced by a wider trend where research studies will have to use the measures used in clinical practice.

There are several reasons why there might be disagreement between the GRS and the changes in the depression and anxiety scales. For example, someone might report feeling better because their anxiety symptoms have improved rather than their depressive symptoms. We have carried out a study to look at this and other factors (Hobbs et al., 2020). In this paper, we are estimating average MCIDs and the strong association between depression and anxiety should not affect our interpretation of the results. For example, if an intervention leads to an improvement of anxiety symptoms more than depressive symptoms, this should be reflected in the treatment effect exceeding the MCID for anxiety but not depressive symptoms. Therefore, calculating separate MCIDs for the different scales will help their use clinically.

Individual expectation is an important determinant of a patient's view of their condition, and new medication prescription could raise expectations. However, conversely, it is also true that a prescription of longer duration that fails to produce a benefit can lead to lower expectations. In this study, most of the patients had already been prescribed antidepressants (88%). We do not have data to distinguish those with a newly prescribed medication. Even if such data were available, much larger sample sizes would be required to examine their impact on MCID estimates. Furthermore, in our study, we were primarily interested in retrospective judgements about change rather than future expectations about improvement.

There are many factors that may influence an individual patient's views of improvement. We have previously reported that patients with more severe anxiety are less likely to report improvement even after controlling for depressive symptoms (Hobbs et al., 2020). In principle, factors that might influence MCID could be incorporated as interaction effects in our model for change in self-rated scores and, if present, these could impact on MCID estimates. However, more individualised MCIDs would require a substantially larger sample size to have the power to detect any interactions.

### Implications

We provide different MCID estimates for depression and anxiety scales that are widely used in both primary care and psychological services. These estimates could help clinicians interpret the relevance of changes in the instrument scores they use. They can be used for an individual patient to estimate changes that are likely to be beneficial. The MCID can be used to compare treatments when accurate predictions of the patients’ future outcomes are available. This way of using the MCID rests on a counterfactual argument in which the future potential outcome of a patient on an active treatment can be contrasted with their future outcome on a comparator. Our MCID estimates could be considered as the minimum difference and allow clinical relevance to be determined in relation to the treatment effects and their confidence intervals from RCTs and meta-analyses. It is also important that the power of clinical trials is sufficient to detect the MCID. The MCID enables clinicians to interpret the effectiveness of treatments in RCTs in primary care beyond statistical significance.

In our previous paper (Button et al., 2015) we suggested that the MCID for the BDI-II was about 17.5%. At the moderate and higher severities in this study, we again find that roughly a 20% change for the PHQ-9, BDI-II and GAD7 corresponded to an MCID. However, at lower severities, this was no longer the case. Unsurprisingly perhaps, patients needed larger percentage changes at low scores to be able to endorse improvement. Our results indicate that even if treatment effects are similar in those with less severe symptoms, it is much less likely that these patients will experience a benefit. This also implies that treatment effects from RCTs may overestimate the effectiveness of treatment for patients with mild symptoms.

The analysis here is based upon the patient identifying an improvement in symptoms retrospectively. This is a slightly different idea than that of ‘clinical importance’ underlying the MCID concept. It seems reasonable to conclude that if someone has not noticed improvement there has not been a clinically important benefit. In contrast, it is possible someone has noticed an improvement, but this is not necessarily of clinical importance. In that sense, our approach provides a minimum above which treatment effects can be judged.

There is currently much controversy about the benefits or otherwise of antidepressant treatment, especially in those with less severe symptoms. Our approach provides a basis on which to power clinical studies and help clinicians interpret the evidence and judge whether a treatment is likely to benefit a patient treated in primary care. More empirical studies considering patients’ perspectives and using large databases of electronic health records are needed to refine MCIDs in specific decision contexts alongside predictions of patients’ outcomes. Different MCIDs are required to support different decisions to initiate, monitor or discontinue treatment whilst accounting for individual patient characteristics as well as patient preference.

## Supplementary material

For supplementary material accompanying this paper visit https://doi.org/10.1017/S0033291720003700. click here to view supplementary material
