# Supplementary material for: How much change is enough? Evidence from a longitudinal study on depression in UK primary care
Source: Psychol Med. 2020 Nov 3;52(10):1875–82. doi: 10.1017/S0033291720003700 (PMC9340848; doi:10.1017/S0033291720003700)
Supplement: Supplementary file 1 [file S0033291720003700sup.zip › S0033291720003700sup001.docx]

**Appendix 1**

**Beta Regression Model**

Beta regression modelling can have substantial advantages when outcomes are bounded and exhibit high levels of skewness. These include substantial improvements in fit as well as increased precision for individual predictions. Beta regression also models outcomes on a multiplicative scale.

More importantly, beta regression allows us to simultaneously explore covariate effects not only on our expectations but also variability which is important for the receiver operating curve (ROC parameters) estimation. The quantification of variability is very often un-appreciated and selectively reported if at all. This state of affairs is despite its importance in sample size calculations required for the design of RCTs as well as meta-analytic studies. Recent methodological advances have allowed a more widespread use of generalised location/scale modelling such as mixed effects beta regression through standard statistical software and for more complex settings e.g. repeated measures analyses.

There is a difference between the regression model we used and a binary regression models where the outcome is whether the patient reports an improvement as a function of the change in their depression scores. The former regression model assumes that the expected value of outcome score change depends on the patient’s view of their condition whereas the later assumes that the expected value of the patient’s view of how they feel depends on their change in scores of BDI. For this reason, we based the estimation of the required ROC parameters on the regression model we described in the previous section.

Each of the outcomes Y=PHQ9, BDI-II and GAD-7 all of which are bounded within (a,b), where a=0 and b=27 for PHQ9; b=63 for BDI-II and b=21 for GAD-7 We transformed the scale to (0,1) interval by applying the transformation Ynew=(Y-a)/(b-a)

The reparameterization used for modelling the mean and variance of the beta distribution follows Ferrari and Cribari-Neto (Ferrari & Cribari-Neto, 2004), had already appeared in the literature (Verkuilen J & M, 2012; Zimprich, 2010).

where i indexes individuals and j indexes visits with j=1,2,3,4

Where is the conditional mean and can be interpreted as a precision parameters, in the sense that for fixed values of the mean larger value of correspond to larger values of the variance for the outcome

The variance is given by:

The parameter represent the change between successive visits and is specific to each CIS-R group and GRS group (log-odds for increase in the outcome). Similarly the parameter represents the baseline values and is specific to each CIS-R group and GRS group

Note, that both intercepts and slopes in this models are also indexed by individuals and these are assumed to be jointly distributed as bivariate Normal distribution with mean zero and a 2X2 variance-covariance, that is also estimated.

Below, we provide the derivations used to compute change on the original scale and percentage change for each group as a function of the model parameters.

Denoting odds parameter (:=exp( ) as

Then the translation to change on the original scale and proportionate change relative to the groups baseline pi is a function the estimated odds and the baseline as follows:

where pi are the outcome values on the transformed ((0-1)) scale.

**MCID determination**

Let and denote the mean of the diagnostic outcome: BDI change (log-ratio) at the gold standard disease status (not feeling better) and feeling better respectively and additional covariates X.

denote the variances of the outcome for the non-diseased (those feeling better) and diseased groups.

The ROC parameters are:

and

Then the Area under the curve A is

The area under the curve is equal to the probability that the outcome for a randomly drawn diseased subject is higher than the randomly drawn non-diseased individual. (represents the standard cumulative normal density)

The sensitivity at given specificity is : Fo any given (1-specificity), p, the underlying sensitivity is:

Finally the Maximum improvement of sensitivity over chance (Youden index, Figure A1): This is the maximum difference in observed sensitivity and sensitivity at change (lying on a 450 line in ROC space) over all values of specificity. The corresponding (1-specificity) denoted by pYOUDEN is given by:

| **Table S1.1: Uncertainty estimates surrounding the ROC performance characteristics of the MCID on the PHQ9, BDI-II and GAD-7.** | | | | | | | | | | |
| --- | --- | --- | --- | --- | --- | --- | --- | --- | --- | --- |
|  |  | **Specificity** | | |  | | **Sensitivity** | | | |
| **Outcome** | **Baseline**  **CIS-R** | **Median** | **95% CI** | |  | | **Median** | | **95% CI** | |
| PHQ9 | 0-11 | 0.78 | (0.61 | 0.85) |  | 0.35 | | (0.27 | | 0.49) |
|  | 12-19 | 0.58 | (0.32 | 0.75) |  | 0.54 | | (0.35 | | 0.76) |
|  | 20+ | 0.63 | (0.49 | 0.73) |  | 0.53 | | (0.42 | | 0.65) |
| BDI-II | 0-11 | 0.80 | (0.69 | 0.85) |  | 0.36 | | (0.30 | | 0.45) |
|  | 12-19 | 0.65 | (0.42 | 0.77) |  | 0.50 | | (0.37 | | 0.69) |
|  | 20+ | 0.65 | (0.50 | 0.75) |  | 0.51 | | (0.41 | | 0.65) |
| GAD-7 | 0-11 | 0.79 | (0.54 | 0.86) |  | 0.33 | | (0.25 | | 0.53) |
|  | 12-19 | 0.51 | (0.33 | 0.67) |  | 0.63 | | (0.45 | | 0.78) |
|  | 20+ | 0.56 | (0.37 | 0.72) |  | 0.54 | | (0.37 | | 0.73) |

In Table S1.1, we depict the uncertainty surrounding the ROC performance characteristics of the MCID and considerable uncertainty is apparent. This uncertainty when propagated leads to uncertainty for the optimal threshold. This is considerable relative to the apparent differences between MCID estimates at different baseline severity levels.

| **Table S1.2: Baseline SD and SD estimates for change on the PHQ9, BDI-II and GAD-7 for the group reporting feeling better** | | | | | | | | | |
| --- | --- | --- | --- | --- | --- | --- | --- | --- | --- |
|  | **Feeling Better** | | | | | **Feeling The Same** | | | |
| **Outcome** | **Baseline**  **CIS-R** | **SD**  **Baseline** | **95% CI** | **SD**  **Change** | **95% CI** | **SD**  **Baseline** | **95% CI** | **SD**  **Change** | **95% CI** |
| PHQ9 | 0-11 | 2.60 | (2.203.06) | 3.35 | (2.863.89) | 2.52 | (2.162.93) | 3.24 | (2.81 3.70) |
|  | 12-19 | 2.08 | (1.79 2.43) | 2.68 | (2.35 3.07) | 2.50 | (2.18 2.86) | 3.21 | (2.86 3.60) |
|  | 20+ | 3.60 | (2.91 4.24) | 4.64 | (3.80 5.40) | 3.01 | (2.66 3.40) | 3.87 | (3.50 4.25) |
| BDI-II | 0-11 | 4.92 | (4.04 5.90) | 6.21 | (5.21 7.31) | 4.05 | (3.40 5.09) | 5.08 | (4.39 5.85) |
|  | 12-19 | 3.69 | (3.15 4.25) | 4.64 | (4.04 5.26) | 3.76 | (3.24 4.72) | 4.71 | (4.12 5.39) |
|  | 20+ | 6.09 | (4.88 7.42) | 7.68 | (6.24 9.15) | 4.50 | (3.76 5.65) | 5.64 | (4.82 6.53) |
| GAD-7 | 0-11 | 2.33 | (1.90 2.82) | 3.04 | (2.51 3.64) | 2.33 | (1.97 2.71) | 3.02 | (2.61 3.47) |
|  | 12-19 | 1.90 | (1.63 2.21) | 2.48 | (2.15 2.84) | 2.45 | (2.14 2.80) | 3.18 | (2.81 3.60) |
|  | 20+ | 3.43 | (2.76 4.03) | 4.48 | (3.69 5.14) | 2.54 | (2.06 2.99) | 3.30 | (2.71 3.84) |

| **Table S1.3: Standardized change over time for PHQ9, BDI-II and GAD-7 according to patient reported Global ratings and baseline CIS-R** | | | | | | | | | | | |
| --- | --- | --- | --- | --- | --- | --- | --- | --- | --- | --- | --- |
| GRS | **Feeling Better** | | |  | **Feeling Same** | | |  | **Feeling Worse** | | |
|  | **Mean** | **95% CI** | |  | **Mean** | **95% CI** | |  | **Mean** | **95% CI** | |
| Baseline CIS-R | **Standardised to Change (Change per 2 weeks/SD Change) for PHQ9** | | | | | | | | | | |
| <11 | -0·30 | -0·42 | -0·19 |  | -0·10 | -0·18 | -0·03 |  | 0·16 | 0·03 | 0·29 |
| 12-19 | -0·51 | -0·69 | -0·35 |  | -0·26 | -0·42 | -0·10 |  | 0·12 | -0·05 | 0·29 |
| 20+ | -0·52 | -0·64 | -0·40 |  | -0·15 | -0·24 | -0·06 |  | 0·06 | -0·03 | 0·16 |
|  | **Standardised to Change (Change per 2 weeks/SD Change) for BDI-II** | | | | | | | | | | |
| <11 | -0·48 | -0·62 | -0·36 |  | -0·28 | -0·36 | -0·20 |  | 0·01 | -0·10 | 0·13 |
| 12-19 | -0·67 | -0·86 | -0·50 |  | -0·35 | -0·51 | -0·19 |  | -0·02 | -0·18 | 0·14 |
| 20+ | -0·60 | -0·76 | -0·46 |  | -0·24 | -0·35 | -0·14 |  | 0·02 | -0·08 | 0·12 |
|  | **Standardised to Change (Change per 2 weeks/SD Change) for GAD-7** | | | | | | | | | | |
| <11 | -0·27 | -0·39 | -0·17 |  | -0·11 | -0·18 | -0·04 |  | 0·19 | 0·06 | 0·33 |
| 12-19 | -0·50 | -0·67 | -0·35 |  | -0·17 | -0·30 | -0·04 |  | -0·00 | -0·14 | 0·14 |
| 20+ | -0·32 | -0·41 | -0·23 |  | -0·09 | -0·16 | -0·02 |  | 0·14 | 0·06 | 0·22 |

**References**

Ferrari, S., & Cribari-Neto, F. (2004). Beta Regression for Modelling Rates and Proportions. *Journal of Applied Statistics, 31*(7), 799-815. Retrieved from <https://EconPapers.repec.org/RePEc:taf:japsta:v:31:y:2004:i:7:p:799-815>

Verkuilen J, & M, S. (2012). Mixed and mixture regression models for continuous bounded responses using the beta distribution. *J Educ Behav Stat, 37*(1), 82-113.

Zimprich, D. (2010). Modeling change in skewed variables using mixed beta regression models. *Res Hum Dev, 7*(1), 9-26.
