# Supplementary material for: How much change is enough? Evidence from a longitudinal study on depression in UK primary care
Source: Psychol Med. 2020 Nov 3;52(10):1875–82. doi: 10.1017/S0033291720003700 (PMC9340848; doi:10.1017/S0033291720003700)
Supplement: Supplementary file 1 [file S0033291720003700sup.zip › S0033291720003700sup002.docx]

**Appendix 2: Figures**

Figure S2.1 Distribution of response to the Global Rating of Change Scale over time

*Figure S2.2a: Distribution of change in PHQ9 scores for those reporting “Feeling better” and those reporting “Feeling the same” according to baseline CIS-R strata with the MCID depicted.*

*Figure S2.2b: Distribution of change in BDI-II scores for those reporting “Feeling better” and those reporting “Feeling the same” according to baseline CIS-R strata with the MCID depicted.*

*Figure S2.2c: Distribution of change in GAD-7 scores for those reporting “Feeling better” and those reporting “Feeling the same” according to baseline CIS-R strata with the MCID depicted.*
