# Supplementary material for: How much change is enough? Evidence from a longitudinal study on depression in UK primary care
Source: Psychol Med. 2020 Nov 3;52(10):1875–82. doi: 10.1017/S0033291720003700 (PMC9340848; doi:10.1017/S0033291720003700)
Supplement: Supplementary file 1 [file S0033291720003700sup.zip › S0033291720003700sup003.docx]

**Appendix 2: Tables**

| **Table S2.1: Baseline characteristics** | |
| --- | --- |
| **Characteristic** | **N (%) unless stated otherwise** |
| Sex |  |
| Female | 273 (68.3) |
| Male | 127 (31.8) |
| Ethnic group |  |
| White | 391 (97.8) |
| Ethnic minority | 9 (2.3) |
| Highest qualification |  |
| Non-compulsory (A Level or above) | 254 (64) |
| Compulsory or below  (GCSE equivalent or no qualifications) | 146 (36) |
| Financial status |  |
| Comfortable | 92 (23) |
| Doing alright | 130 (32) |
| Just about getting by | 109 (27) |
| Finding it difficult | 69 (17) |
| Marital status |  |
| Married/cohabiting | 212 (53) |
| Single | 101 (25) |
| Separated/divorced/widowed | 87 (22) |
| Currently taking antidepressants |  |
| Yes | 288 (72) |
| No | 111 (28) |
| Taken antidepressants in the past |  |
| Yes | 350 (88) |
| No | 50 (12) |
| Age, Mean (SD) | 48.7 (12.5) |
| BDI-II score, Mean (SD) | 19.7 (11.8) |
| PHQ-9 score, Mean (SD) | 10.1 (6.7) |
| CIS-R score, Mean (SD) | 9.3 (5.6) |

| **Table S2.2: Distribution of responses to the Global Rating of change scale over time** | | | | | |
| --- | --- | --- | --- | --- | --- |
|  |  | **Occasion** | | | |
| **Global** | **Rating** | **Baseline** | **Visit 1** | **Visit 2** | **Visit 3** |
| Feeling | Better | 93 | 121 | 142 | 133 |
|  | (%) | (23.25) | (30.25) | (35.5) | (33.25) |
|  | Same | 253 | 186 | 155 | 187 |
|  | (%) | (63.25) | (46.5) | (38.75) | (46.75) |
|  | Worse | 54 | 93 | 103 | 80 |
|  | (%) | (13.5) | (23.25) | (25.75) | (20) |
|  | **Total** | 400 | 400 | 400 | 400 |

The proportion of people reporting feeling the same reduced over time from 63.3% at baseline to 46.8% at the third visit (Table A2.2, Appendix 2). These reductions were due to increases in the proportion of those who reported feeling either better or worse with the most dramatic changes occurring at the first visit. The proportion reporting feeling better increased from 23.3% at baseline to 30.3% during the first visit and remained at similar or slightly higher levels for the remainder of follow-up. The proportion reporting feeling worse also increased from 13.3% at baseline to 23.3% during the first visit and also remained at similar or slightly higher levels for the remainder of follow-up.

At baseline 46.3% (n=185) had a CIS-R score of 20 or higher and 31% (n=123) had CIS-R levels below 12 points. Among those with a CIS-R score of 20 or higher, the majority (63%, n=117) reported feeling the same and 17% (n=32) reported feeling better compared with the two weeks previously. Among those with moderate (n=78) and low CIS-R score (n=58) a majority of 63% reported feeling the same Among those with low CIS-R score 29% (n=36) reported feeling better. Among those with moderate CIS-R 27% (n=25) reported feeling the same (Figure A2.1, Appendix 2: Figures).
